# Stable Operation of Copper-Protected La(FeMnSi)_13_H_*y*_ Regenerators in a Magnetic Cooling Unit

**DOI:** 10.1021/acsaenm.4c00747

**Published:** 2025-01-13

**Authors:** Nico P. Weiß, Ulysse Rocabert, Cornelia Hoppe, Jens-Peter Zwick, Konrad Loewe, Maximilian Fries, Antti J. Karttunen, Oliver Gutfleisch, Falk Muench

**Affiliations:** † Magnotherm Solutions GmbH, Pfungstädter Straße 102, 64297 Darmstadt, Germany; ‡ Inorganic Materials Modelling Group, Department of Chemistry and Material Science, Aalto University, FI-00076 Aalto, Finland; § Functional Materials, Institute of Materials Science, Technische Universität Darmstadt, Peter-Grünberg-Straße 16, 64287 Darmstadt, Germany; ∥ Vacuumschmelze GmbH & Co. KG, Grüner Weg 37, 63450 Hanau, Germany

**Keywords:** alloys, functional materials, coatings, electroless plating, copper, corrosion, stability, magnetic cooling

## Abstract

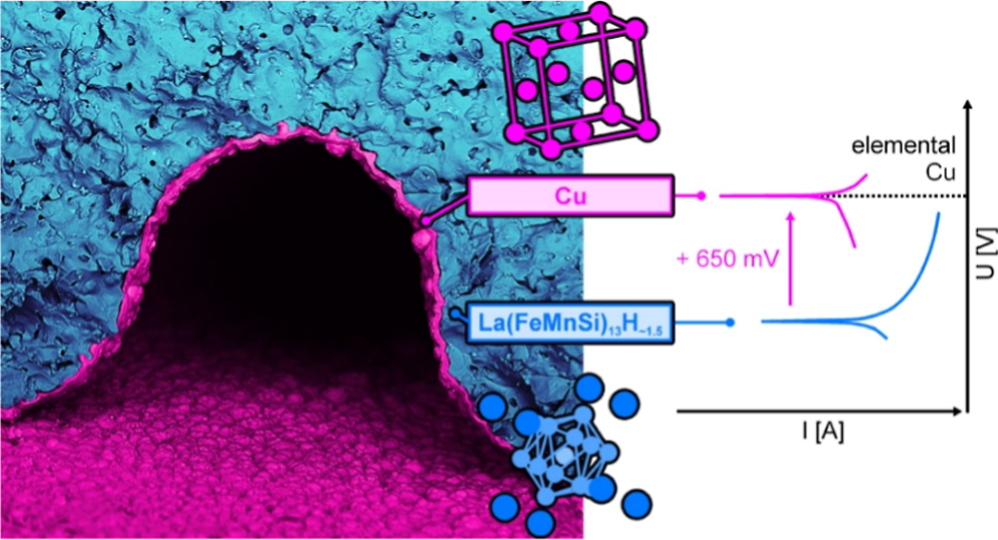

Magnetic refrigeration leads the current commercialization efforts of ambient caloric cooling technologies, is considered among its peers most promising in terms of anticipated energy efficiency gain, and allows for complete elimination of harmful coolants. By now, functional magnetocaloric components (so-called regenerators) based on Mn-substituted and hydrogenated LaFeSi alloys are commercially available. However, this alloy system exhibits magnetostriction, is susceptible to fracture, oxidation, and does not passivate well, rendering it prone to failure and corrosion, particularly when using water as favorable heat exchange medium. Demonstrating stable and extended operation of LaFeSi-based regenerators under realistic conditions with cost-sensitive measures thus constitutes a key milestone for derisking the materials system, paving a path toward reliable and maintenance-friendly magnetic cooling devices. Building upon a fundamental analysis of materials properties, process, and target specifications, we outline a 2-fold protection strategy, encompassing a highly conformal copper coating working in tandem with a tailored inhibitor system. The former is applied using an optimized electroless plating procedure, allowing us to evenly envelop complex regenerator architectures in a dense, nondefective, homogeneous, and ductile copper film of excellent interfacial quality. The latter addresses the corrosion characteristics of both coating and substrate in the application environment. In-device aging experiments prove the effectiveness of our multifaceted approach in maintaining the chemical, mechanical, and functional integrity of LaFeSi regenerators under continuous use.

## Introduction

Rising average global temperatures, urbanization, increasing demand for perishable foods, and rising incomes are driving the importance of cooling technologies to new heights. Already, nearly 20% of global energy is used by the cooling sector, with projections to increase immensely by the end of the century.^1^ Satisfying this demand will become one of the most significant challenges but also a chance in the cooling industry for the next decades. Being established almost two centuries ago—the first working system was constructed in 1834^2^—vapor-compression refrigeration systems still constitute the currently dominant technology for ambient cooling. Due to the limitations of its underlying thermodynamic cycle^3,4^ and its maturity, further efficiency gains are increasingly difficult to realize. Gas compressors traditionally depend on halogenated hydrocarbons as fluid refrigerants, which exhibit a high global warming potential and, in the case of the already phased-out chlorinated compounds, deplete the ozone layer. Regulatory pressure toward fluorinated compounds, like the increasingly strict handling of so-called eternity chemicals (PFAS) by the European Union,^5^ drives the implementation of alternative refrigerants such as isobutane, ammonia, or CO_2_ which, however, exhibit trade-offs such as flammability, corrosivity, toxicity, or high-pressure demands.^6−8^

To overcome these process-intrinsic issues, novel means of generating a temperature gradient are required. Caloric cooling technologies based on highly reversible thermodynamic cycles can simultaneously achieve increased energy efficiencies and make harmful refrigerants obsolete.^9−12^ Currently, magnetic refrigeration represents the most developed caloric cooling technology, is considered among its peers particularly promising in terms of anticipated energy efficiency gain,^4,9,13,14^ and runs particularly well when combining the solid magnetocaloric refrigerants with the benign heat exchange medium water,^15^ which offers favorable physicochemical properties such as low viscosity, high heat capacity, and thermal conductivity aside being inexpensive and harmless.

The advent of performant and cost-efficient magnetocaloric materials systems operable at ambient temperatures has brought the technology to the brink of market entry. While gadolinium still dominates the prototyping landscape of ambient magnetic cooling,^15^ its ability to supply the expansive cooling market is hampered by its cost and criticality,^16^ and alternative materials with improved scaling characteristics are in active development. By now, functional magnetocaloric elements—so-called regenerators—based on Mn-substituted and hydrogenated alloys derived from the La(FeSi)_13_ intermetallic^17^ are commercially available^18,19^ (approximate sum formula La(Fe_13–*x*–y_Mn_*x*≤0.4_Si_1.2<*y*<1.5_)_13_H_1.5≤*z*_, abbreviated below as “LaFeSi” for convenience). Such alloys show a magnetocaloric effect in a remarkably wide temperature span (typical Curie temperatures range from approximately 135–200 K for the unhydrogenated and 280–345 K for fully hydrogenated samples^20^ due to the shifting effects of hydrogen-induced lattice expansion and modulation of the magnetic environment by Mn.

While tunable, highly reversible, performant, and composed of sufficiently abundant elements,^4,20,21^ these LaFeSi alloys are susceptible to mechanical fracture (the intermetallic nature of the magnetocaloric phase and the hydrogenation process renders the material highly brittle) and chemical attack.

LaFeSi alloys are oxophilic and corrode in water, even if it is deoxygenated and deionized,^22^ showing a behavior resembling their majority constituent Fe, which is typically present in amounts of 75–80 m % but rendered more ignoble due to the presence of La.^23^ They do not passivate well, rendering them highly prone to corrosion in contact with aqueous heat exchange media.^24−26^ To promote heat transfer, magnetocaloric regenerators are finely structured and percolated by continuous pores or pore networks. Typical material thicknesses and hydraulic pore diameters lie in the range of several 100 μm.^27,28^ The resulting large, exposed regenerator surface area amplifies the corrosion issues of the ignoble and chemically sensitive LaFeSi alloys.^24^ Rust formation is observed during in-device use,^28^ resulting in ongoing loss of active material and release or accumulation of corrosion products, which can block regenerator pores and damage hydraulic components. Given the pronounced reliability demands and cost sensitivity of target markets such as food refrigeration, a clear demonstration of the long-term stability of LaFeSi-based regenerators in their application environment constitutes a prerequisite for their use in maintenance-friendly magnetic cooling devices and thus represents a key milestone in derisking the materials system. Efforts to stabilize and shape LaFeSi powders by bonding with polymers^29^ or low-melting alloys^30^ are suffering from the low thermal conductivity of polymers and issues related to the processing and composition of low-melting metals (e.g., use of critical elements such as In, difficult wetting), and weaknesses of the obtained composite materials (e.g., remaining porosity, incomplete coverage, increased thermal load, limited utilization of binder embedded within the composite). Combining LaFeSi particles sputter-coating with Cu prior to forming a compacted composite with plasma spark sintering showed inconsistent Cu film quality, did not provide suitable regenerator architectures (notably, embedding regular, narrow, dense, and continuous pore arrays for flushing) and only resulted in a slight potential shift in linear sweep voltammetry (LSV) measurements, indicating only a minor increase in corrosion protection.^31^

Corrosion studies on LaFeSi alloys typically focus on traditional methods such as mass loss or LSV,^22,25,32^ which do not properly replicate the conditions during operation of a magnetocaloric cooling device. These are, for instance, the dynamic flow conditions during bidirectional flushing of the regenerators, the use of heat exchange media instead of model electrolytes, long exposition times, magneto-transport,^33^ or the structural change accompanying the phase transition of LaFeSi magnetocaloric’s.^34^ As a result of the repeated (de)magnetization during operation, LaFeSi alloys breathe, which can exacerbate degradation phenomena such as depassivation and microcracking during use. Therefore, in-device tests are paramount for obtaining reliable stability data and for verifying specific protection strategies. One such realistic aging experiment has been performed with uncoated LaFeSi regenerators, which showed visible but nondestructive corrosion after 5 million cycles.^18^

In this study, we build on our fundamental stability analysis of Mn-substituted and hydrogenated LaFeSi alloy^24^ and show that by combining a highly conformal, micrometer-thin metal coating shielding the sensitive substrate material against direct attack with an inhibitor mixture tailored to our specific materials system, regenerator oxidation and mechanical failure alike can be prevented under operating conditions for extended periods of time.

## Results and Discussion

LaFeSi exhibits poor passivation properties, which cannot be easily overcome by compositional optimization. Elements like Ni or Cr, which are typically employed in considerable quantities to improve the corrosion stability of Fe, diminish the magnetocaloric effect and shift the transition to second order in the case of Ni,^35^ and increase the irreversibility of the magnetocaloric transition before a similar second order shift in the case of Cr.^36^ The limitations of increasing the intrinsic resilience of the alloy and the inability of hitherto explored inhibitors to completely prevent corrosion^24^ motivate coating strategies to create a protective interface while maintaining the functionality of the bulk alloy.

Such efforts are demanding both in terms of the required coating properties and process characteristics. The coating material must be ductile and endure the cyclic volume change of the regenerator without crack formation, possess a high thermal conductivity to not hamper the crucial heat transfer between the regenerator and water, and be stable under operation conditions. The employed process must apply the coating in a thin, dense, and homogeneous fashion (notably across the large and difficult-to-access inner surface area of the regenerator) to reliably seal the susceptible LaFeSi surface without clogging fluid channels, introducing superfluous thermal mass and attacking the susceptible material. This rules out methods that create thick, uneven, or nonconformal deposits, rely on elevated temperatures at which hydrogenated LaFeSi alloys suffer from hydrogen and function loss,^37^ or utilize corrosive environments. Given the substantial amounts of magnetocaloric materials required to serve the extensive cooling market, the method must also be scalable and cost-efficient (e.g., in an advanced prototype, 1.52 kg LaFeSi alloys were used for generating ∼2.5 kW cooling power).^38^

Metal coatings in the micrometer thickness range can fulfill all previously mentioned demands. Due to its outstanding conformality and industrial applicability,^39^ electroless plating is uniquely suited for homogeneously depositing such films on the intricate magnetocaloric regenerator architectures at scale, avoiding shadowing effects that appear with the use of other thin-film application processes. Our protection strategy utilizes Cu as a coating material, which we chose due to its outstanding thermal conductivity (401 W/mK,^40^ compared to 6.0 W/mK of pure LaFeSi^41^), ductility, relative nobility, and frequent use in electroless plating. A suitable electroless deposition procedure must quickly overcoat the sensitive LaFeSi surface while suppressing undesired side reactions such as corrosion, uncontrolled galvanic displacement, and homogeneous nucleation. Furthermore, it must ensure high plating rates and the reliable formation of dense, nondefective coatings. For this purpose, we developed a deposition procedure addressing the specific reactivity of LaFeSi regenerators (patent pending),^42^ the results of which are outlined below. The unique suitability of our optimized deposition procedure is corroborated by the failure of different commercial Cu deposition protocols, including electroless Cu plating with and without Pd seeding, as well as Cu electrodeposition, to achieve convincing results (see Supporting Information, Figure SI1,2).

**Figure 1:**
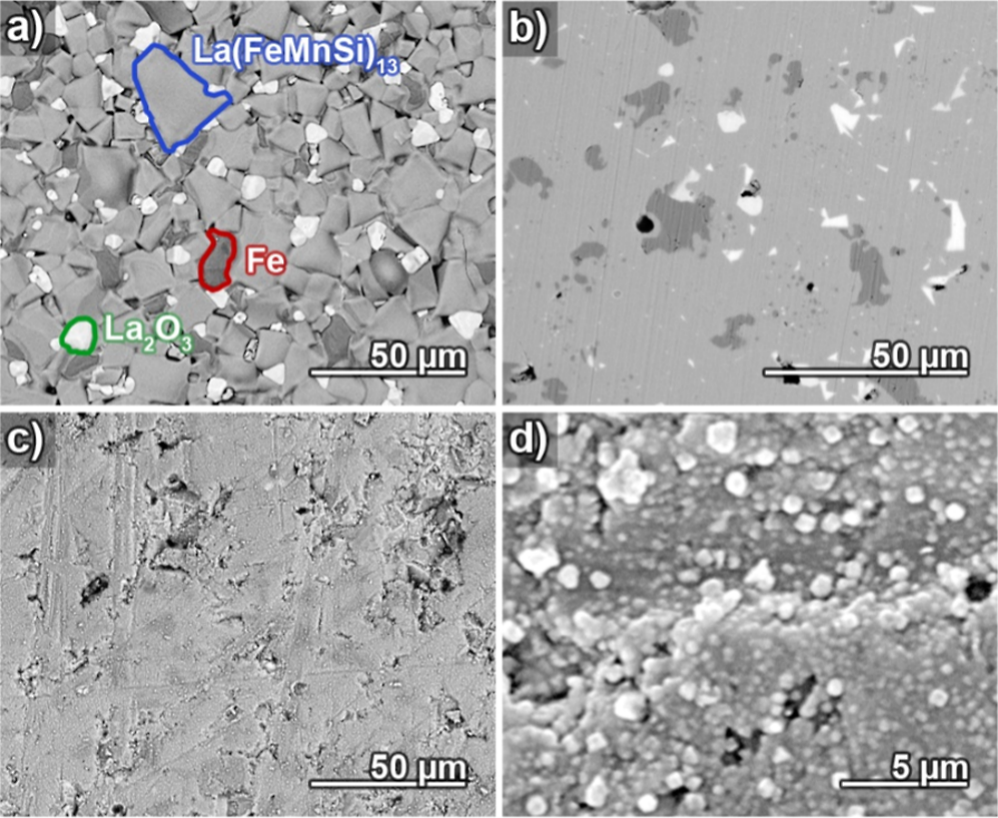
Top view electron micrographs of a LaFeSi plate showing (a) the sintering skin with highlighted grains of the present main phases La(FeMnSi)_13_ (medium gray), α-Fe (dark gray) and La_2_O_3_ (light gray), composition verified by EDX spectroscopy, (b) the same material, but mechanically polished, (c) polished material subjected to 3 min of electroless Cu plating, (d) a magnified image of the plated surface shown in (c).

The initial stages of coating formation were investigated by comparing pristine and briefly plated LaFeSi substrates with scanning electron microscopy (SEM, Figure 1). All LaFeSi substrates employed in this study were prepared by sintering precursor powders. Their microstructure is dominated by the magnetocaloric active La(FeMnSi)_13_ phase but also contains α-Fe precipitates (a slight Fe excess is introduced to improve the mechanical stability of the alloys) and minor quantities of La-rich phases such as La_2_O_3_, which represents a preferred oxidation product and can be found in increased amounts on the sintering skin (Figure 1a). The plates were mechanically polished to provide an even and reproducible surface for plating and the subsequent electrochemical corrosion test. Besides the three phases mentioned before, a small extent of internal porosity in the μm size range is evident as dark spots in the polished plates (Figure 1b), a remnant of the powder metallurgical origin of the materials.

The Cu deposition is initiated by simply immersing the LaFeSi samples into the plating solution. The reaction onset is indicated by hydrogen gas evolution, which is accompanied by a color change of the gray LaFeSi substrate to the metallic rose of the forming Cu film (eq 1)
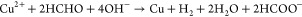
1

Once Cu deposits are formed, the reaction is carried on by autocatalysis: The reaction product—metallic Cu—is catalytically oxidizing the present formaldehyde,^43^ releasing electrons for the continuous reduction of complexed Cu(II) ions. This autonomous nature of the reaction is the mechanistic foundation for its ability to produce coatings of outstanding homogeneity, even on extended interior surfaces such as present in magnetocaloric regenerators. Also, if dominant, this surface-catalyzed pathway confines the reaction to the substrate-liquid interface and ensures the selective metallization, impeding the disruptive side reaction of homogeneous nucleation, in which the spontaneous reduction and precipitation of metal in the bulk solution results in the uncontrolled formation of metal powders.^39^

However, the reliance on a heterogeneously catalyzed reaction pathway poses a challenge for initially inactive substrates. As such, common electroless plating protocols employ seeding steps to outfit inactive substrates with catalyst particles (e.g., Ag or Pd^39^). We tuned our bath composition to enable spontaneous Cu deposition on LaFeSi substrates, rendering additional seeding steps obsolete. This simplifies the deposition procedure, reduces cost, and protects the sensitive LaFeSi substrates from attack by, e.g., acidic sensitization or oxidizing noble metal solutions used for seeding.^39^ Given the pronounced standard potential difference between Cu and our LaFeSi alloy, we expect a spontaneous galvanic displacement reaction to be responsible for the initial formation of superficial Cu seeds, which quickly merge to form a closed, continuously growing Cu layer. Already after 3 min of plating, the clear phase pattern and smooth surface of the polished sample (Figure 1b) disappeared, and the complete substrate was evenly covered by a particulate Cu film (Figure 1c,d), hinting at a high density and even distribution of spontaneously formed Cu seeds. Both the absence of visible oxidic residues and the retention of the substrate topography (apart from the deposition of Cu particles) indicate a controlled coating buildup without pronounced substrate attack by either corrosion or galvanic displacement. Interestingly, we found that using a surfactant significantly reduced the time from sample insertion into the plating bath to the start of the plating reaction and improved bubble detachment. We attribute this to the better interface of the sample and plating bath due to the introduction of a surfactant. Additionally, using a surfactant decreased the operational window regarding temperature and pH, allowing for a more stable plating bath without a risk of homogeneous nucleation.

**Figure 2:**
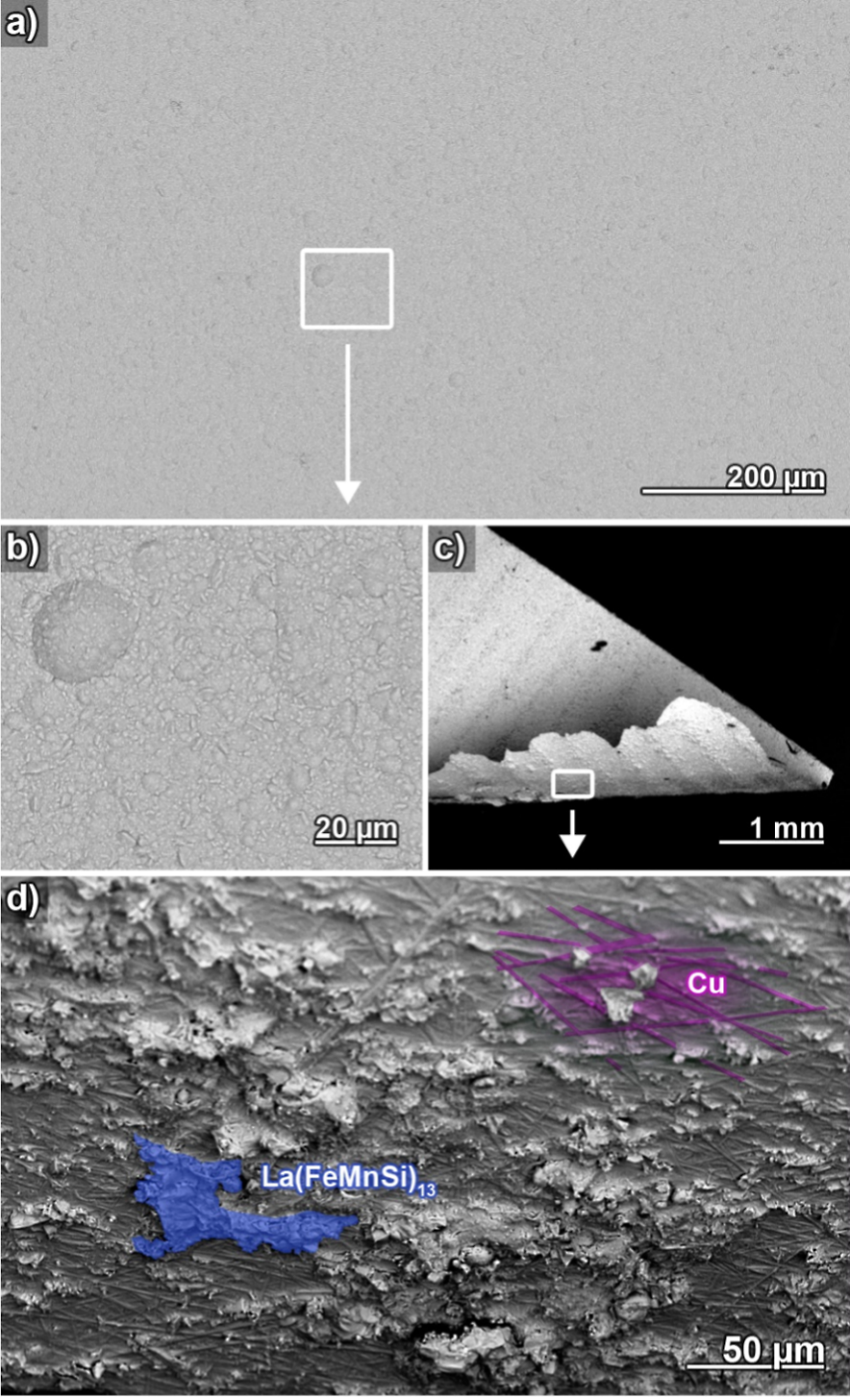
Electron micrographs of a Cu film deposited on a LaFeSi plate for 2 h. (a) Top view image of the film at low magnification. The marked region is magnified in (b), which details the microstructure of the evolved coating. (c) Survey image of a peeled-off Cu film. The marked region at the bottom of the detached coating is magnified in (d), in which Cu, as well as debris from the underlying LaFeSi substrate, are highlighted.

**Figure 3:**
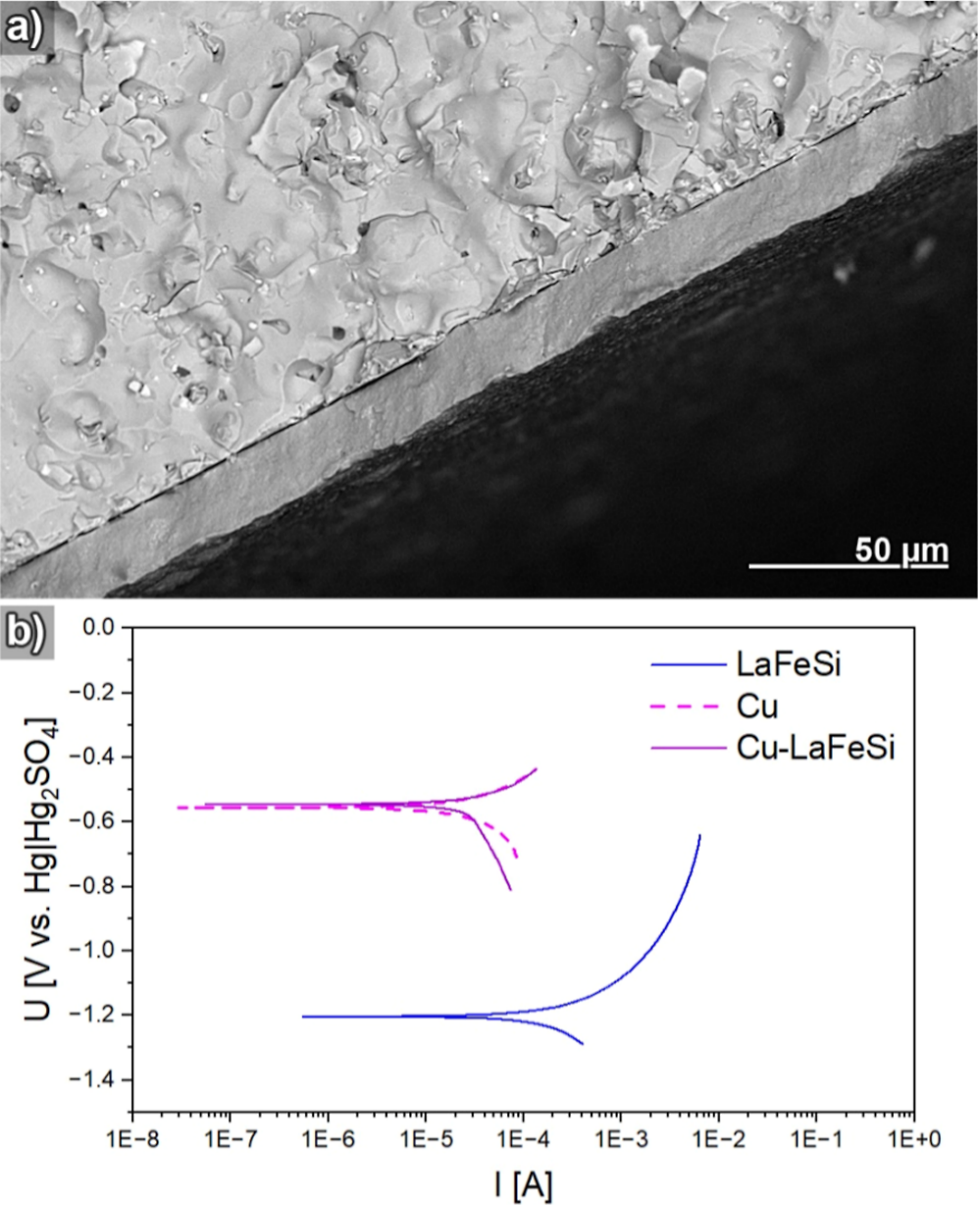
(a) Electron micrograph of a breaking edge of a Cu-coated LaFeSi plate. (b) LSV of Cu-coated LaFeSi plate and pure LaFeSi as well as Cu as reference materials, measured in buffered electrolyte (pH ∼ 8.2, 100 mM tris(hydroxymethyl)aminomethane +20 mM citric acid).

To assess the coating and interface quality in more detail, LaFeSi plates were subjected to an extended plating time of 2 h (Figure 2). The coating is dense and exhibits excellent homogeneity on a large scale, without notable defects (Figure 2a). The surface structure evolved from the initial polished (Figure 1b) and early deposition state (Figure 1c) and now exhibits an altered, generally smooth, but somewhat grainy microstructure representative of the developed Cu film (Figure 2b). When breaking the sample and forcefully peeling off the Cu film (Figure 2c), LaFeSi fragments remain embedded underneath the Cu layer (Figure 2d, blue highlight). This observation implies an efficient mechanical interlocking between Cu and LaFeSi, e.g., at positions where the Cu deposits fill up substrate pores. Apparently, the ultimate strength of the coating layer is defined by the brittle fracture of the substrate. No clean delamination of the Cu film is observed, indicating good adhesion. Additional top-view SEM characterization of the Cu film bottom can be found in the Supporting Information (Figure SI3). On the bottom of the Cu film, straight ridges are found (Figure 2d, magenta highlight). These structures represent inverse replicas of the scratches stemming from mechanical polishing (see grooves in Figure 1b) and are evidence of the tight contact between substrate and coating. A cross-sectional analysis of the Cu-coated LaFeSi sample corroborates the absence of oxidation products as well as the density, interlocking, homogeneity, and completeness of the coating (Figure 3a). Based on cross-sectional images and mass gain experiments, typical maximum plating rates of 6–8 μm h^–1^ are found, which somewhat decrease with increasing plating time due to reagent depletion (see Supporting Information, Figure SI4). Such plating rates match industry standards^44^ and enable a sufficiently quick deposition of protective coatings, which are expected to adopt thicknesses between a few μm and a few tens of μm.

LSV was employed to chemically interrogate the nature of the solution-accessible surface of the Cu-coated LaFeSi plate, which provides a direct measure for the achieved degree of protection (Figure 3b). Aside from the coated sample, pure LaFeSi and pure Cu references representing the boundary cases of the mixed materials system were measured to allow for a quantitative assessment of the coating-induced corrosion potential shift. A rather negative corrosion potential of −1.2 V was obtained for pure LaFeSi, which matches its electropositive constituent elements and clearly contrasts the −0.55 V found for the seminoble Cu reference. Coating the LaFeSi caused a marked positive shift of its corrosion potential by +0.65 V and made it coincide with the Cu reference. Also, the anodic branches of both the Cu-coated LaFeSi and the Cu reference overlapped and showed considerably reduced corrosion current densities as compared to the anodic branch of the LaFeSi reference. As such, the electrochemically probed interface of the coated sample very closely resembles that of pure Cu, corroborating the completeness and low defect level of the coating as seen in SEM (Figures 2a,b and 3a). Previous efforts to coat LaFeSi substrates with protective Ni–P coatings resulted in smaller potential shifts and anodic branches whose initial current densities compared similarly to pure LaFeSi.^45^

**Figure 4:**
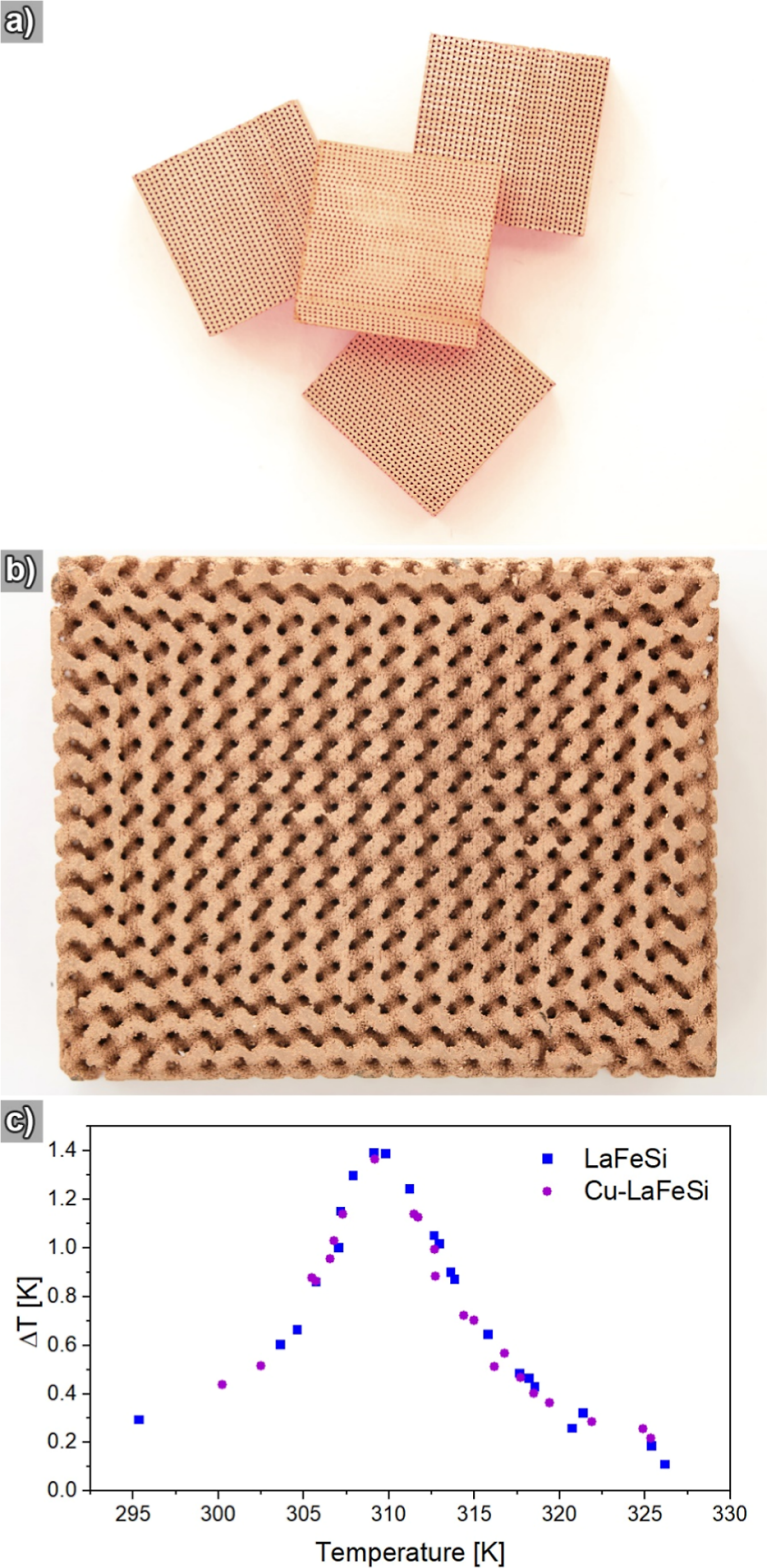
(a) Photograph of stacked, Cu-coated LaFeSi microchannel regenerators. (b) Photograph of a Cu-coated freeform LaFeSi regenerator produced by binder jetting. (c) Characterization of the magnetocaloric effect of coated and pristine microchannel regenerators, measured as adiabatic temperature change at 1 T over the magnetic transition temperature range.

Finally, we applied our deposition reaction to commercial LaFeSi-based microchannel regenerators (CALORIVAC),^19^ porous blocks composed of stacked wavy layers and enclosing hundreds of straight and parallel pores per cm^2^. The as-obtained regenerators were evenly metalized and displayed the bright shine of elemental Cu (Figure 4a). We want to highlight that our plating protocol is applicable to even more complex shapes, such as demonstrated in an initial experiment with a regenerator produced by three-dimensional (3D) printing (Figure 4b), a technology whose freeform capabilities provide appealing synergies in conjunction with electroless plating.^46^ Importantly, their magnetocaloric function did not deteriorate during plating (Figure 4c), which we attribute to the modest reaction temperature (<60 °C) and the gentle bath chemistry. Apparently, the ductile and thin Cu coating did not impose a considerable enough strain on the LaFeSi substrate to affect the magnetocaloric transition by confining the material during magnetostriction.^47^ Other novel strategies to improve corrosion resistance and thermal conductivity by utilizing graphene cannot provide sufficient protection without sacrificing magnetocaloric performance.^48^

**Figure 5:**
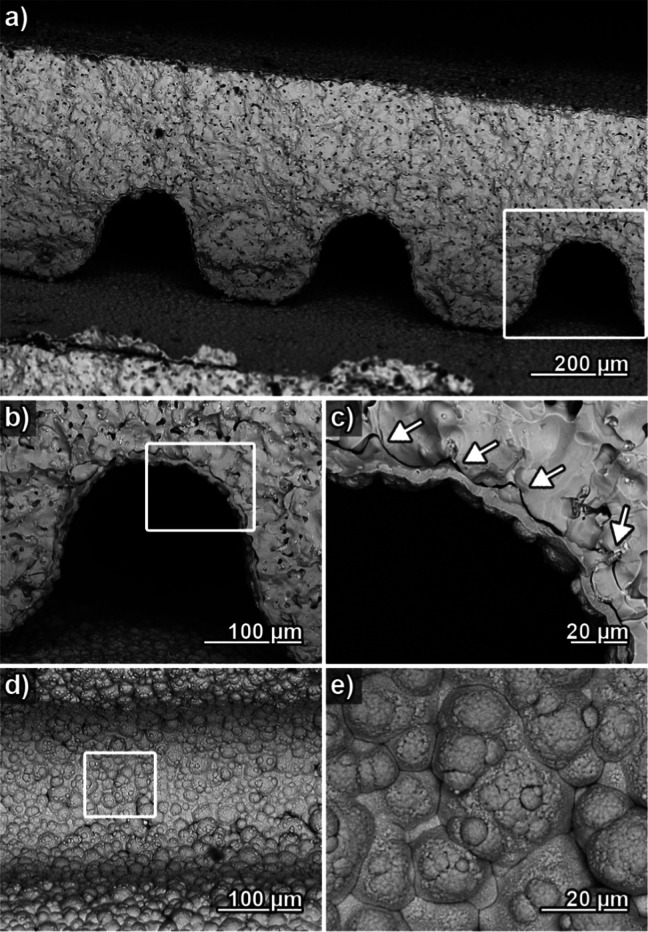
(a) Electron micrograph focusing on a single layer of a Cu-plated, fractured microchannel regenerator. (b) Magnified image of the individual channel marked in (a). (c) Magnified image of the wall section marked in (b), with arrows highlighting the Cu coating filling up porous features of the rough LaFeSi surface. (d) Top view image of a Cu-coated channel. (e) Magnified image of the channel bottom area marked in (d).

SEM analysis of coated microchannel regenerators verified homogeneous Cu deposition across their interior surfaces (Figure 5). An animated image showcasing the evolution of a microchannel layer from uncoated to coated, drafted using identical location SEM, can be found in the Supporting Information (Figure SI5). All key features the coating displayed on planar LaFeSi substrates (mechanical interlocking, high density, interface tightness and cleanliness, low defect level) could be successfully transferred to the complex regenerator shape (Figure 5a–c), although the coating appears much rougher due to the increased roughness of the underlying substrate, which is closely reproduced by the deposition reaction (Figure 5d,e).

To investigate the efficacy of our corrosion protection strategy, we implemented Cu-coated and pristine LaFeSi microchannel regenerators in magnetic refrigeration devices under realistic conditions, exposing them to different aqueous heat exchange fluids optionally containing corrosion inhibitors with bidirectional flushing and repeated (de)magnetization. For the following in-device aging experiments, we compared deionized water as a reference with two inhibitor mixtures: a previously reported commercial formulation based on the suspected carcinogen 4-nitrobenzoate,^49^ which was chosen due to its ability to efficiently protect both Cu and cast Fe, materials resembling our (coated) regenerators, and an in-house inhibitor mixture developed to protect both Cu and LaFeSi without resorting to toxic chemicals compromising safe and convenient device maintenance. Keeping the combined materials system in mind is crucial since galvanic coupling between Cu and LaFeSi can increase the oxidative pressure at regions where the coating is wounded. The employed inhibitor formulation must prevent both meaningful erosion and marked oxidation of the Cu coating and simultaneously stabilize the chemically quite different LaFeSi substrate. For instance, during static inhibitor screening tests with submerged Cu–LaFeSi microchannels broken into layers to create defects prone to galvanic corrosion, formulations often were capable of either protecting Cu or LaFeSi (see Supporting Information, Figure SI6).

**Figure 6:**
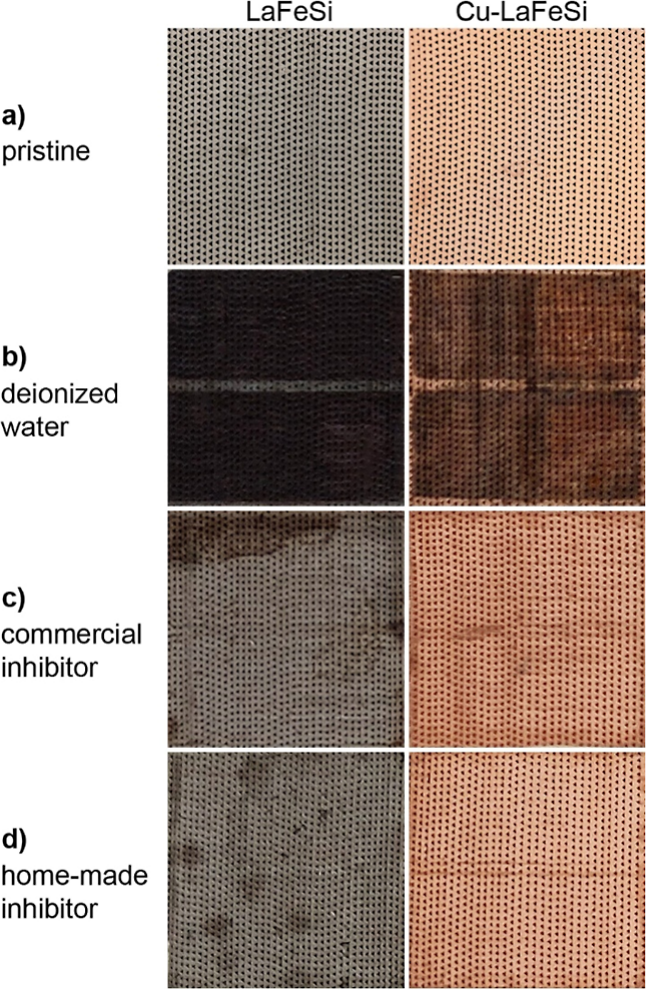
Photographs of uncoated and copperized LaFeSi regenerators (a) before implemented in the test devices. (b) After a six-week period working with deionized water (c) after a six-week period working with a commercial inhibitor mixture (d) after a six-week period working with a homemade inhibitor mixture. The line visible for every picture in the middle of the regenerators is caused by a sample holder, preventing direct contact of the regenerator with the respective heat-exchanging fluids at this position.

The stability results shown below were obtained by cycling the regenerators in the test devices for 6 weeks, using one Cu-coated and one pristine regenerator each (Figure 6). An optical comparison of copperized and uncoated regenerators before (Figure 6a) and after (Figure 6b–d) their use in the test devices shows evident changes in color, explainable by corrosion processes, which are especially pronounced in the case of the deionized water reference (Figure 6b). Here, both the coated and uncoated regenerators are darkened where they were in contact with the heat-exchanging fluid, and reddish particles were found in the water stream. The pronounced oxidation of the LaFeSi regenerator in this chemically gentle medium demonstrates the ample need for corrosion protection in this vulnerable magnetocaloric materials system.

Copperized regenerators working with the two inhibitor mixtures show nearly no change in color; the regenerator exposed to the commercial inhibitor mixture appears slightly darker. While uncoated regenerators show an apparent oxidation reduction compared to the pure water reference, they are not unharmed (Figure 6c,d). The uncoated regenerator operated in the commercial inhibitor mixture shows a color change that indicates rust formation at the upper left edge in the picture (Figure 6c), and the uncoated regenerator operated in the homemade inhibitor mixture shows spots of darkened material (Figure 6d). Already, this optical first glance makes it evident that a protection strategy merely employing an inhibitor mixture is struggling to reliably prevent corrosion processes on LaFeSi during operation. The combined effect of inhibitor and coating leads to very good results, with only a minor darkening of the Cu surface in the case of the commercial formulation (Figure 6c), and an almost unaltered appearance in the case of our self-developed formulation (Figure 6d).

**Figure 7:**
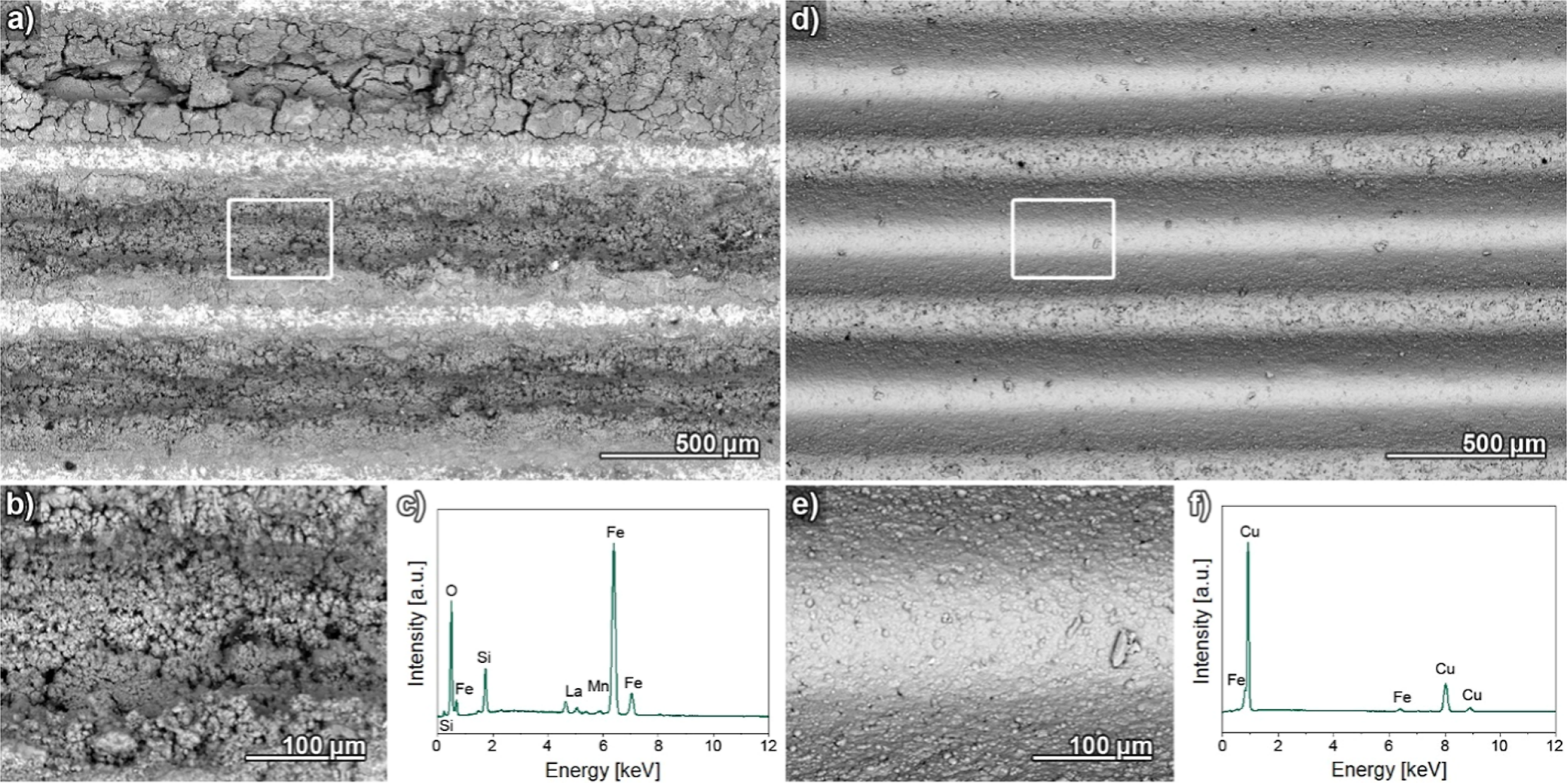
(a) Electron micrograph showing multiple, nearly clogged channels of an uncoated LaFeSi regenerator working with deionized water. (b) Magnified image of the porous, spongy material responsible for clogging the channel area marked in (a). (c) EDX graph of the area shown in (b) confirming rust formation due to the detection of iron oxides. (d) Electron micrograph showing multiple channels of a copperized LaFeSi regenerator working with the homemade inhibitor mixture. (e) Magnified image of the position marked in (d), showing a single copperized channel bottom without signs of corrosion products. (f) EDX graph of the area shown in (b) confirming a pristine copper surface without oxygen detection.

To build upon the optical characterization, SEM/energy-dispersive X-ray (EDX) analysis was used for a corrosion analysis with increased spatial resolution and added elemental information. These measurements confirmed the rust formation assumed based on the regenerator color change. In case of the uncoated channel operating in deionized water, SEM analysis reveals a porous, spongy, poorly adhering layer formed on the inner surfaces of the channels, which significantly decreases their diameter and increases the pressure drop this regenerator produces in the cooling cycle (Figure 7a,b). Letting this test device run even longer than 6 weeks would cause a near-complete clogging of the channels, while expelled rust particles could block or damage of other components in the hydraulic cycle. EDX analysis of the respective areas confirms these structures to be mainly iron oxides with additional C contaminations (Figure 7c). Similar micrographs of a copperized regenerator working with deionized water are shown in the Supporting Information (Figure SI7a–c). Here, the immense amount of corrosion products found in the uncoated regenerator is absent, but large amounts of O are detected alongside particles on top of the Cu coating, which likely stem from both Cu oxidation and sedimented oxides transported here from the uncoated regenerator by the fluid stream. Likewise, micrographs of an uncoated regenerator working with the homemade inhibitor mixture are shown in the Supporting Information (Figure SI7d–f). While significant amounts of O could be detected with EDX, SEM shows no porous structure such as the ones visible after operation in deionized water. Apparently, the inhibitor mixture can passivate the LaFeSi surface to significantly slow down but, according to the photograph, cannot wholly prevent corrosion processes (Figure 6d). In agreement with the visual impression of the aged sample, the commercial inhibitor mixture resulted in a present but minor oxidation of LaFeSi and a very slight oxidation of the Cu layer (see Supporting Information, Figure SI8).

The SEM/EDX analysis confirmed the excellent impression of the Cu-coated sample operated in our in-house inhibitor. Electron micrographs show a pristine, smooth and dense surface that is undistinguishable from before the device implementation (Figure 7d,e). The complete absence of cracks provide proof that the applied Cu coating can accommodate the magnetic breathing the LaFeSi regenerator experiences. EDX confirms the well-preserved state of the Cu coating surface, with no detectable amounts of O (Figure 7f).

## Conclusions

Proving the stable operation of LaFeSi regenerators is imperative for derisking this vital caloric materials system for ambient magnetic cooling. Based on the fundamental corrosion properties of the material and key processing and functionality requirements of the technology, we rationally developed and implemented a scalable, 2-fold protection strategy. First, a seed-free electroless plating reaction is used to apply a dense and homogeneous Cu coating to all surfaces of the complex-shaped regenerators without clogging the fluid channels, damaging the substrate, compromising its function or imposing meaningful thermal barriers while shielding the sensitive material from solution access and enduring its magnetic breathing without fracture. Second, inhibitor-augmented aqueous heat exchange media are employed which preserve the Cu coating and reduce corrosive attack on the potentially exposed LaFeSi substrate as much as possible. When applied in conjunction, these measures protect application-ready LaFeSi components under realistic operation conditions with unprecedented efficacy. After 6 weeks of continued operation, the regenerators are almost undistinguishable from their prior state. Applying the results gained in this work, the service life of LaFeSi-based regenerators in refrigeration systems can be significantly increased, decreasing the need for costly replacements and maintenance. Preliminary results of the ongoing experiments show the preservation of the pristine microchannel state after 3 months (see Supporting Information, Figure SI9).

Based on our promising findings, we will pursue long-term experiments lasting several months to years to demonstrate maintained performance at time scales approaching the product life cycle. Such experiments also should investigate varying coating thicknesses to strike a good balance between the improved protective capabilities of thick coatings on one hand and associated disadvantages such as increased plating time and cost, thermal mass and heat transfer across the regenerator on the other. Apart from increasing the coating thickness, a second coating layer or altered inhibitor formulations could be used to adjust the corrosion characteristics.

Cu-coated regenerators show a considerably increased overall mechanical stability and are less prone to fracture. This warrants more detailed mechanical investigations as well as metallurgical studies trying to translate the robustness added by the Cu coating to a reduced Fe excess in the LaFeSi alloy, with the aim of shifting the trade-off between mechanical stability and performance more in the direction of improved function.

Finally, the ability of autocatalytic Cu deposition reactions to coat intricate substrate shapes makes our approach compatible with metal 3D printing, opening avenues for the fast, tool-free adjustment and optimization of the LaFeSi architecture and a small to midscale production of protected regenerators, an area we actively explore.

## Materials and Methods

### Materials and Chemicals

Following chemicals were used without additional purification: 1,2,3-benzotriazole (Carl Roth, ≥99% for synthesis), benzoic acid sodium salt (Carl Roth, ≥99% Ph. Eur.), citric acid monohydrate (Carl Roth, >99%), sodium hydroxide (Carl Roth, ≥99% bead form), sodium nitrate (Carl Roth, ≥99% cryst.), tris(hydroxymethyl)aminomethane (Carl Roth, >99%).

Ultrapure water (18.2 MΩ cm), which was obtained by running deionized water through a Milli-Q system, was used for preparing the electrolyte.

Hydrogenated and Mn-substituted LaFeSi microchannel regenerators with the composition La(Fe_13–*x*–*y*_Mn_*x*≤0.4_Si_1.2<*y*<1.5_)_13_H_1.5≤*z*_ were purchased from VACUUMSCHMELZE GmbH & Co. KG for the in-device testing. The coating is also performed on LaFeSi plates of similar stoichiometry and regenerators utilizing a partial substitution of La with Ce (see Figure 5), confirming the ability of our coating to extend to related LaFeSi alloy compositions.

### Coating Procedure

Electroless plating is carried out using formaldehyde (Carl Roth, ≥37%) as a reduction agent. Additionally, a surfactant (e.g., polysorbates, organosulfonates or -sulfates, sodium or potassium soaps), a complexing agent, a Cu-source (ideally CuSO_4_), a stabilizer, a ductility enhancing agent, an accelerator, a buffer, and a pH adjustment agent are used, as described in the patent.^42^ Plating is performed at temperatures below 60 °C at a pH between 12.5 and 13. The temperature prevents hydrogen loss in the magnetocaloric material, whereas the strongly alkaline pH reduces the substrate reactivity (which shows poor passivation in an alkaline environment but quickly dissolves in acids^24^), limiting corrosion side reactions and improving the interface quality.

### Assessment of Magnetocaloric Function

The adiabatic temperature change of the regenerators was investigated using a homemade measurement rig, in which samples are heated to allow measurements (applying a magnetic field of 1 T, measuring the change of sample temperature after this field is applied, and the sample temperature at which this change happens) at different temperatures around the expected Curie temperature of the respective sample and to construct a graph (adiabatic temperature change over sample temperature) likewise to the one shown in this work.

### In-Device Testing

The coated regenerators were inserted into modified POLARIS^50^ devices from MAGNOTHERM Solutions GmbH. The regenerators were subject to a load-free operation while being flushed with different heat exchange media precooled to their Curie temperature (∼288 K) to allow for the induction of magnetic breathing, for which a magnetization frequency of 2 Hz was employed (field strength: ∼0.8 T). As heat exchange media, deionized water, a homemade inhibitor mixture and an inhibitor mixture inspired by a BASF patent^49^ (derived from example 7:20 g/L 4-nitrobenzoic acid, 30 g/L sodium benzoate, 0.5 g/L sodium nitrate, 0.7 g/L benzotriazole, 6 g/L NaOH; borax and sodium silicate were taken out of the formulation to avoid the formation of brittle oxidic layers, ethylene glycol was removed due to the lacking need for antifreeze, and NaOH was added for pH adjustment and solubilization) were employed.

### Scanning Electron Microscopy

For all SEM measurements, a TESCAN VEGA3 is used, which is equipped with an Ametex EDAX octane for EDX measurements. Metal samples for characterization were attached to aluminum holders with carbon tape for characterization. An acceleration voltage of 20 kV is used during imaging. All images are collected using backscattered electrons.

### Linear Sweep Voltammetry

The electrochemical measurements were conducted with a three-electrode setup (counter electrode: platinized Ti mesh; working electrode: planar metal samples; reference electrode: Hg|Hg_2_SO_4_ in 0.5 M H_2_SO_4_) driven by a Gamry 600 potentiostat using a scan rate of 1 mV s^–1^. The working electrodes were prepared by attaching the samples to a Cu strip with carbon tape and paste, followed by insulating the complete surface apart from a window of defined area (∼1.8 cm^2^), which was oriented to face the counter electrode in a parallel fashion, akin to our previous study.^24^ To match the Cu coating on the LaFeSi substrate and eliminate substrate contributions, the Cu reference material was prepared by applying our Cu deposition reaction on an electrochemically inert polycarbonate foil, which was ion-track etched to efficiently anchor the Cu film on the polymer surface,^51^ and seeded with Pd nanoparticles with a two-step sensitization–activation reaction.^52^

## Supporting Information Available

Attempts of Cu deposition using commercial methodologies; bottom view of delaminated Cu coating; plating rate analysis; identical location SEM analysis of Cu coated regenerator layer; inhibitor screening; additional characterization of in-device aged regenerators; SEM analysis of plated regenerator after 3 months aging (DOCX)

## Abbreviations

EDX: energy-dispersive X-ray spectroscopy
LaFeSi: alloys of the approximate stoichiometric composition La(Fe_13–*xy*_Mn_0.2<*x*<0.4_Si_1.2<*y*<1.4_)_13_H_1.5<*z*<1.8_ which exhibit a giant magnetocaloric effect at ambient temperatures and are used throughout this study with an iron excess
LSV: linear sweep voltammetry
SEM: scanning electron microscopy
