# Supplementary material for: Stable Operation of Copper-Protected La(FeMnSi)13Hy Regenerators in a Magnetic Cooling Unit
Source: ACS Appl Eng Mater. 2025 Jan 13;3(1):256–65. doi: 10.1021/acsaenm.4c00747 (PMC11773641; doi:10.1021/acsaenm.4c00747)
Supplement: Supplementary file 1 — em4c00747_si_001.docx [file em4c00747_si_001.docx]

SUPPORTING INFORMATION

Stable Operation of Copper-Protected La(FeMnSi)_13_H_y_ Regenerators in a Magnetic Cooling Unit

Nico Weiß,^1,2,4,*^ Ulysse Rocabert,^2^ Cornelia Hoppe,^2^ Jens-Peter Zwick,^1^ Konrad Loewe,^3^ Maximilian Fries,^1^ Antti Karttunen, ^4^ Oliver Gutfleisch,^1,2^ Falk Muench,^1,*^

1 Magnotherm Solutions GmbH, Pfungstädter Straße 102, 64297 Darmstadt, Germany

2 Functional Materials Group, Department of Materials- and Geosciences, Technische Universität Darmstadt, Peter-Grünberg-Straße 16, 64287 Darmstadt, Germany

3 Vacuumschmelze GmbH & Co. KG, Grüner Weg 37, 63450 Hanau, Germany

4 Inorganic Materials Modelling Group, Department of Chemistry and Material Science, Aalto University, FI-00076, Finland

* Corresponding Authors; e-mail addresses: weiss@magnotherm.com

muench@magnotherm.com.

**TABLE OF CONTENTS**

Attempts of Cu deposition using commercial methodologies ∙∙∙∙∙∙∙∙∙∙∙∙∙∙∙∙∙∙∙∙∙∙∙∙∙∙∙∙∙∙∙∙∙∙∙∙∙∙∙∙∙∙∙∙∙∙∙∙∙∙∙∙∙ 3

Bottom view of delaminated Cu coating ∙∙∙∙∙∙∙∙∙∙∙∙∙∙∙∙∙∙∙∙∙∙∙∙∙∙∙∙∙∙∙∙∙∙∙∙∙∙∙∙∙∙∙∙∙∙∙∙∙∙∙∙∙∙∙∙∙∙∙∙∙∙∙∙∙∙∙∙∙∙∙∙∙∙∙∙∙∙∙∙∙∙∙∙∙ 6

Plating rate analysis ∙∙∙∙∙∙∙∙∙∙∙∙∙∙∙∙∙∙∙∙∙∙∙∙∙∙∙∙∙∙∙∙∙∙∙∙∙∙∙∙∙∙∙∙∙∙∙∙∙∙∙∙∙∙∙∙∙∙∙∙∙∙∙∙∙∙∙∙∙∙∙∙∙∙∙∙∙∙∙∙∙∙∙∙∙∙∙∙∙∙∙∙∙∙∙∙∙∙∙∙∙∙∙∙∙∙∙∙∙∙∙∙∙∙∙∙∙ 7

Identical location SEM analysis of Cu coated regenerator layer ∙∙∙∙∙∙∙∙∙∙∙∙∙∙∙∙∙∙∙∙∙∙∙∙∙∙∙∙∙∙∙∙∙∙∙∙∙∙∙∙∙∙∙∙∙∙∙∙ 8

Inhibitor screening ∙∙∙∙∙∙∙∙∙∙∙∙∙∙∙∙∙∙∙∙∙∙∙∙∙∙∙∙∙∙∙∙∙∙∙∙∙∙∙∙∙∙∙∙∙∙∙∙∙∙∙∙∙∙∙∙∙∙∙∙∙∙∙∙∙∙∙∙∙∙∙∙∙∙∙∙∙∙∙∙∙∙∙∙∙∙∙∙∙∙∙∙∙∙∙∙∙∙∙∙∙∙∙∙∙∙∙∙∙∙∙∙∙∙∙∙∙∙ 9

Additional characterization of in-device aged regenerators ∙∙∙∙∙∙∙∙∙∙∙∙∙∙∙∙∙∙∙∙∙∙∙∙∙∙∙∙∙∙∙∙∙∙∙∙∙∙∙∙∙∙∙∙∙∙∙∙∙∙∙∙∙∙∙ 10

SEM analysis of plated regenerator after 3 months aging ∙∙∙∙∙∙∙∙∙∙∙∙∙∙∙∙∙∙∙∙∙∙∙∙∙∙∙∙∙∙∙∙∙∙∙∙∙∙∙∙∙∙∙∙∙∙∙∙∙∙∙∙∙∙∙ 11

**ATTEMPTS OF CU DEPOSITION USING COMMERCIAL METHODOLOGIES**

As outlined in the main manuscript, the high reactivity of LaFeSi as well as the challenging geometry of the regenerators considerably complicate the successful deposition of thin, dense and homogeneous Cu coatings. A commercial coating provider was contacted and tasked to deposit Cu onto LaFeSi microchannel regenerators using three techniques, namely electroless Cu plating without seeding, electroless Cu plating with Sn/Pd seeding to provide catalyst sites for the reaction, and with Cu electrodeposition.

Without seeding, the commercial electroless plating procedure did not produce any coating. Bath decomposition due to homogeneous nucleation occurred before plating onset, in clear contrast to our reaction, which can initiate Cu deposition without any seeds. Commercial seed-assisted electroless Cu plating produced an inhomogeneous Cu film on the exterior of the regenerator sample (Fig. SI 1 a), which is very rough and most pronounced on exposed sites, quickly decreasing in the amount of deposited Cu with increasing distance from the topmost LaFeSi features reaching into the solution (see Fig. SI 1 b, in which the Fe from the underlying substrate still is dominant, despite the apparent, coral-shaped Cu deposits). When moving from the exterior surface into the microchannels within the regenerator, the weak Cu coating quickly breaks down completely, and only minor Cu amounts are found alongside traces of O (Fig. SI 1 c,d). Interestingly, an etching effect is visible, as visible in the erosion of the medium grey LaFeSi phase, while the dark grey Fe grains rose above the ignoble LaFeSi matrix, which apparently experienced increased dissolution (see Fig. SI 1 c, and compare with the pristine state shown in Fig. 1 a). Commercial Cu electrodeposition resulted in strong corrosive attack of the regenerator surface, with only minor amounts of deposited Cu, which did not provide any meaningful protection (Fig. SI 2).


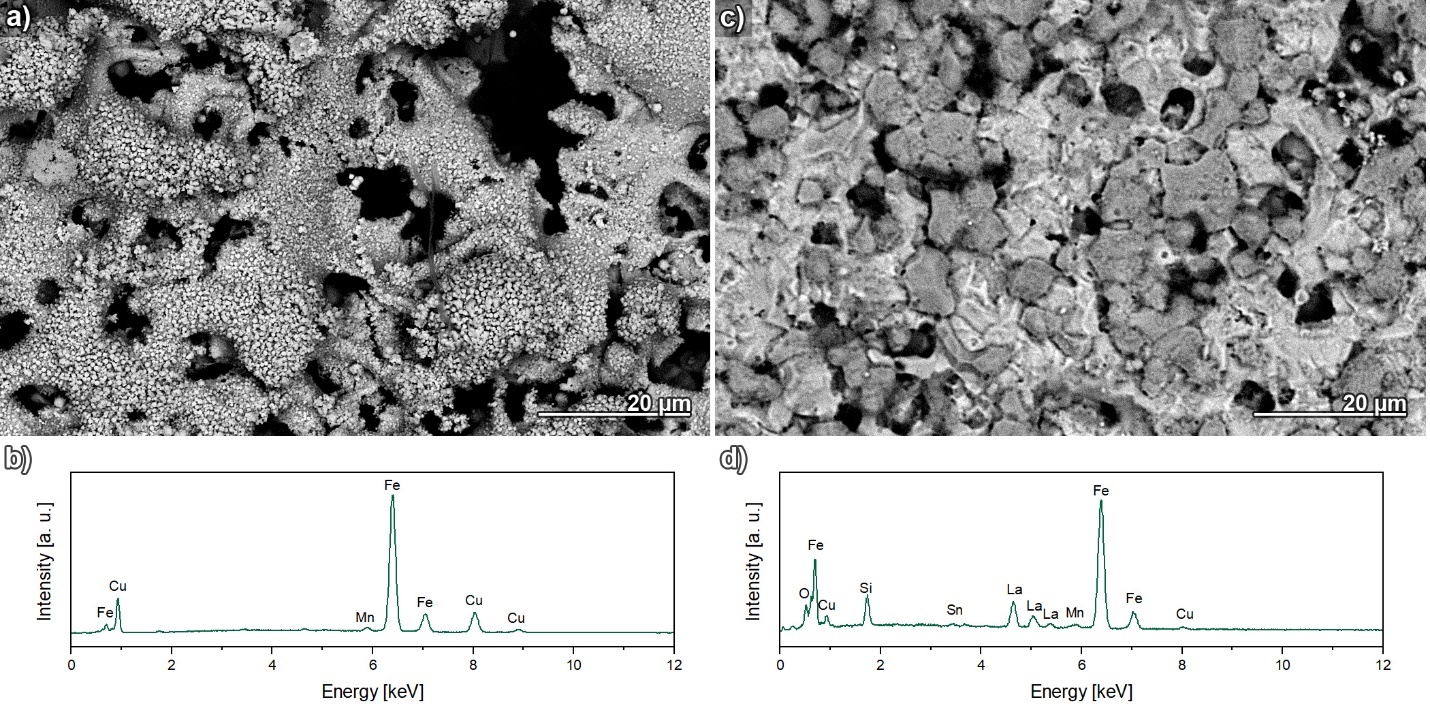


**Fig. SI 1.** SEM/EDX analysis of a LaFeSi microchannel regenerator subjected to a commercial, Sn/Pd-seed-assisted electroless Cu plating procedure, showing exterior (a,b) and interior (c,d) channel surfaces.


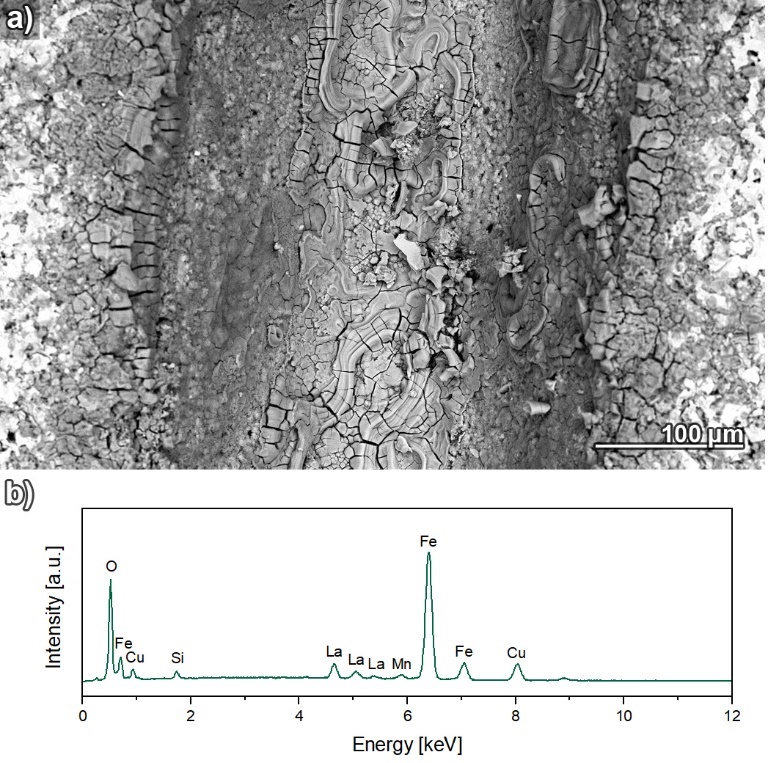


**Fig. SI 2. a)** SEM and **b)** corresponding EDX analysis of a LaFeSi microchannel regenerator subjected to a commercial Cu electrodeposition procedure.

**BOTTOM VIEW OF DELAMINATED CU COATING**

SEM images of the bottom side of the delaminated Cu film show clear features of the excellent inverse replication of the LaFeSi surface features by the coating (prominently, the linear grooves from the polishing step are visible as straight ridges), aside larger chunks of LaFeSi broken out of the substrate.


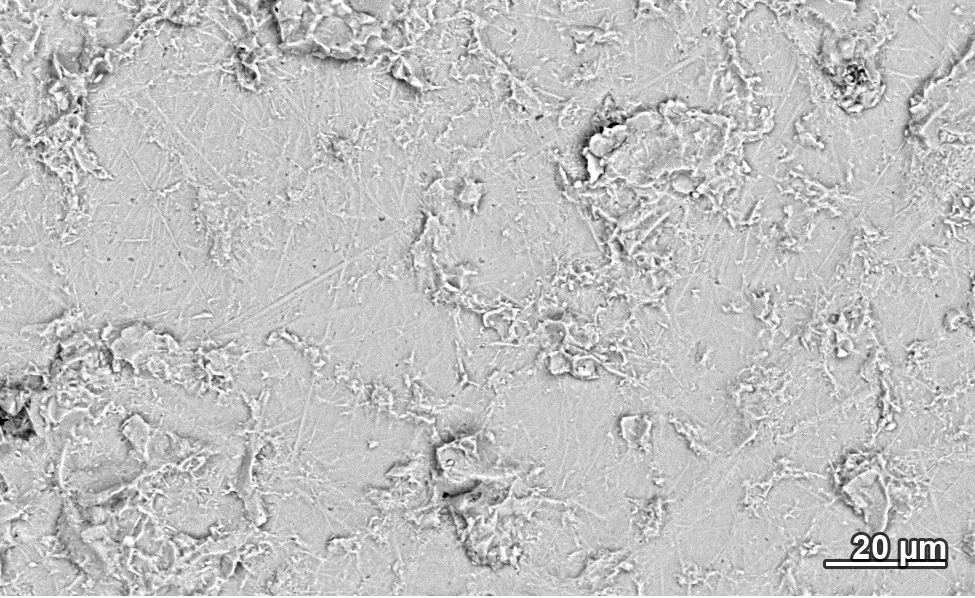


**Fig. SI 3.** SEM image of delaminated Cu coating.

**PLATING RATE ANALYSIS**

The thickness gain of the Cu coating layer over the deposition time was calculated by measuring the mass gain of a LaFeSi plate during plating, under the assumption of a perfectly smooth, planar substrate surface, a constant Cu film thickness and 100% film density. An alternative means of determining the Cu coating thickness was used to verify the indirect mass gain approach (cross-sectional analysis of Cu film by SEM on the fracture edge of coated LaFeSi samples), with well matching results. The thickness gain analysis shows a brief initial induction period corresponding to the nucleation stage of the Cu coating in which the full plating rate is not yet reached, followed by a steady-state regime of roughly linear thickness increase, which slowly declines over time due to reagent depletion and bath aging.


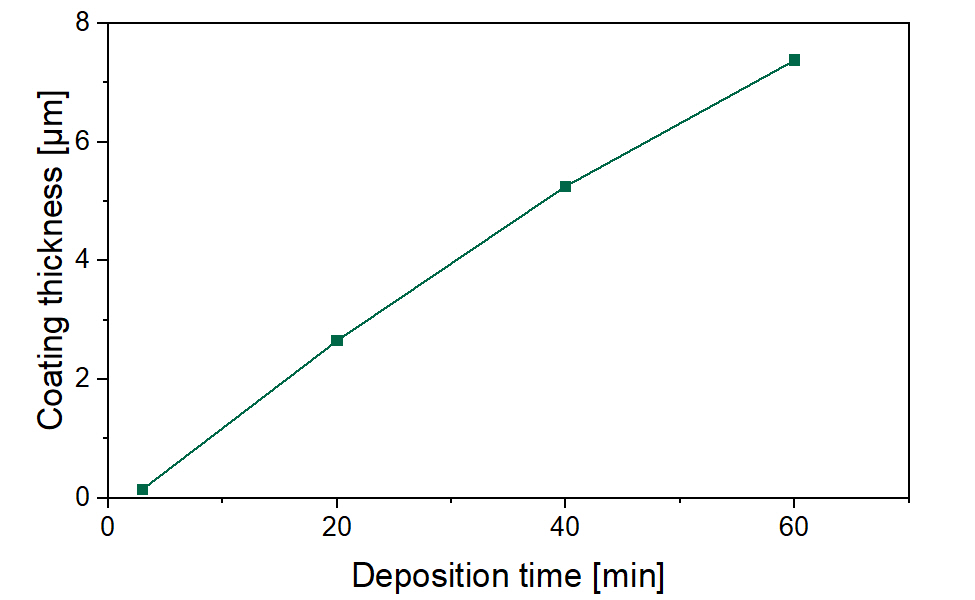


**Fig. SI 4.** Cu coating thickness over plating time, determined by mass gain on a planar sample.

**IDENTICAL LOCATION SEM ANALYSIS OF CU COATED REGENERATOR LAYER**

A microchannel was split into individual layers and characterized by SEM before and after plating. The filling of substrate pores by and the absence of corrosion products on the evolved Cu coating is evident, as is emergence of the typical surface structure of the polycrystalline coating, which covers the complete sample. In the copperized state, fibers are visible primarily at the channel entrance. These structures represent copperized cellulose fibers which attached to and merged with the Cu coating and stem from tissue paper.


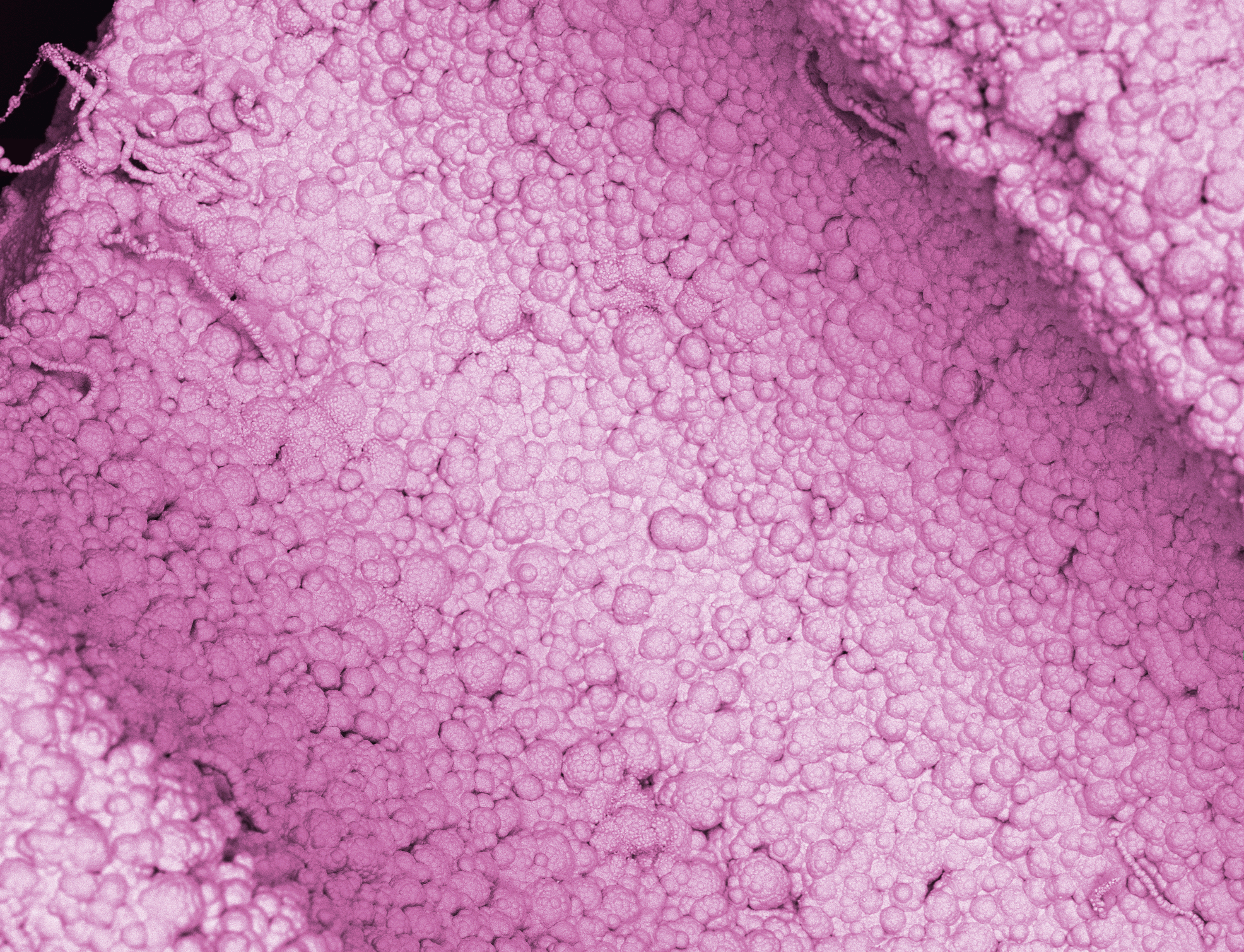


**Fig. SI 5.** Animated image showing the pristine (grey) and plated (pink) surface of a channel.

**INHIBITOR SCREENING**

Before the in-device tests, promising inhibitor formulations were selected based on corrosion screening experiments, which were conducted by immersing regenerator pieces into aqueous inhibitor solutions and optically monitoring their condition while storing them for several weeks. Only formulations capable of properly protecting both the Cu coating and the LaFeSi were chosen for further studies. Achieving simultaneous protection is complicated by the markedly differing corrosion properties of the Cu coating and the LaFeSi substrate. Shown below is an example in which two layers were broken off a Cu-coated microchannel regenerator, exposing pristine LaFeSi surface alongside Cu-coated area to mimic a damaged regenerator and encourage galvanic corrosion. While the specific inhibitor formulation preserved LaFeSi rather well, the Cu coating dissolved over time, as is evident by the blue color of formed Cu(II) complexes.


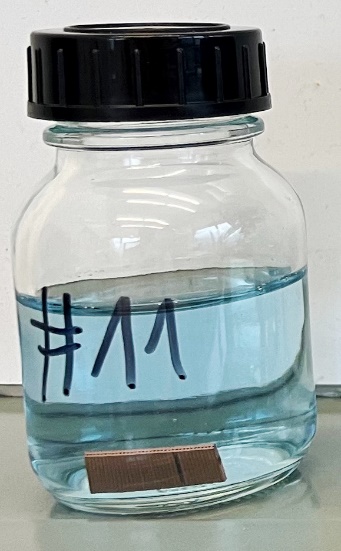


**Fig. SI 6.** Photograph of a static corrosion test showing a dual layer of copperized LaFeSi microchannel immersed in an aqueous inhibitor solution.

**ADDITIONAL CHARACTERIZATION OF IN-DEVICE AGED REGENERATORS**

SEM/EDX analysis reveals the considerable protective capabilities of our Cu coating, which even in the absence of inhibitors remains mostly stable during the six week operation. While some corrosion effects can be found, they mostly are evident in the form of Fe oxides on top of the Cu coating (Fig. SI 7 a-c). This is in accordance with the optical characterization shown in the main manuscript (Fig. 6 b), in which a dark brown deposit of corrosion products is found on a generally well-preserved Cu surface, which can be linked to Fe oxides floating in the heat exchange solution, stemming from the uncoated sample operated in the same device.

Also, the good protective capabilities of our self-developed inhibitor formulation with respect to uncoated LaFeSi was confirmed. After six weeks of operation, the pristine microchannel operated in our in-house mixture only showed minor oxidation, notably without any pore blockage due to the buildup of corrosion products (Fig. SI 7 d-f), in stark contrast to the sample operated in water without inhibitors (Fig. 7 a,b).


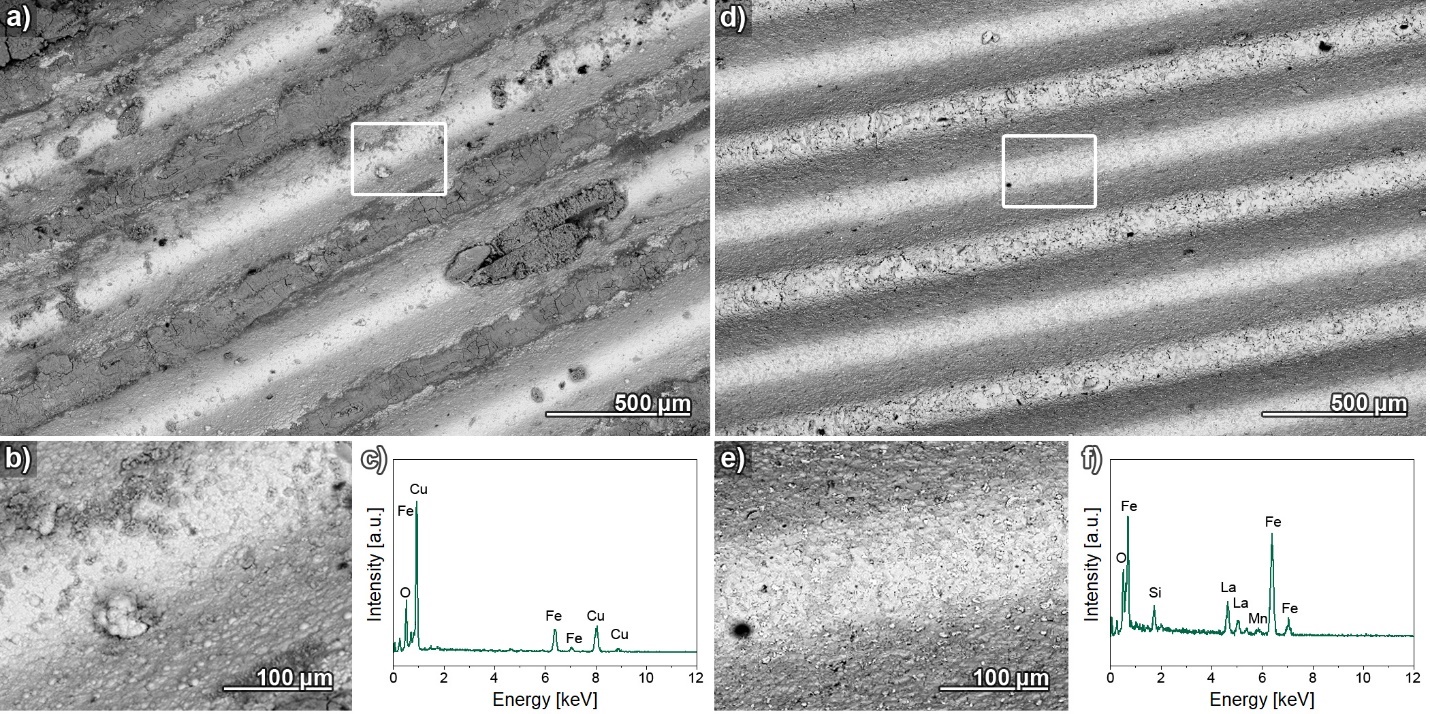


**Fig. SI 7. a)** Electron micrograph showing multiple channels of a coated LaFeSi regenerator operated in deionized water. **b)** Magnified image of a single channel, showing some dark particles of oxidic material on top of the Cu surface. **c)** EDX graph of the area shown in b) confirming the presence of Cu and modest amounts of (Fe)oxides. **d)** Electron micrograph showing multiple channels of a pristine LaFeSi regenerator operated in our home-made inhibitor mixture. **e)** Magnified image of the area highlighted in d), showing a single channel without signs of corrosion products marked in a). **f)** EDX graph of the area shown in b), confirming minor oxidation without the buildup of voluminous corrosion products.

SEM/EDX characterization of the sample operated in the commercially inspired inhibitor mixture (see the samples shown in the main manuscript, Fig. 6 c) is shown in Fig. SI 8. While generally good protection levels are observed, without Cu coating, the buildup of some oxidic products is visible (dark regions in Fig. SI 8 a,b), and a minor oxidation of the substrate surface is found (Fig. SI 8 c). With Cu coating, the regenerator remains structurally unaltered (Fig. SI 8 d,e), though in contrast to the home-made inhibitor (see main manuscript, Fig. 7 e), traces of oxygen were found on the Cu surface (Fig. SI 8 f).


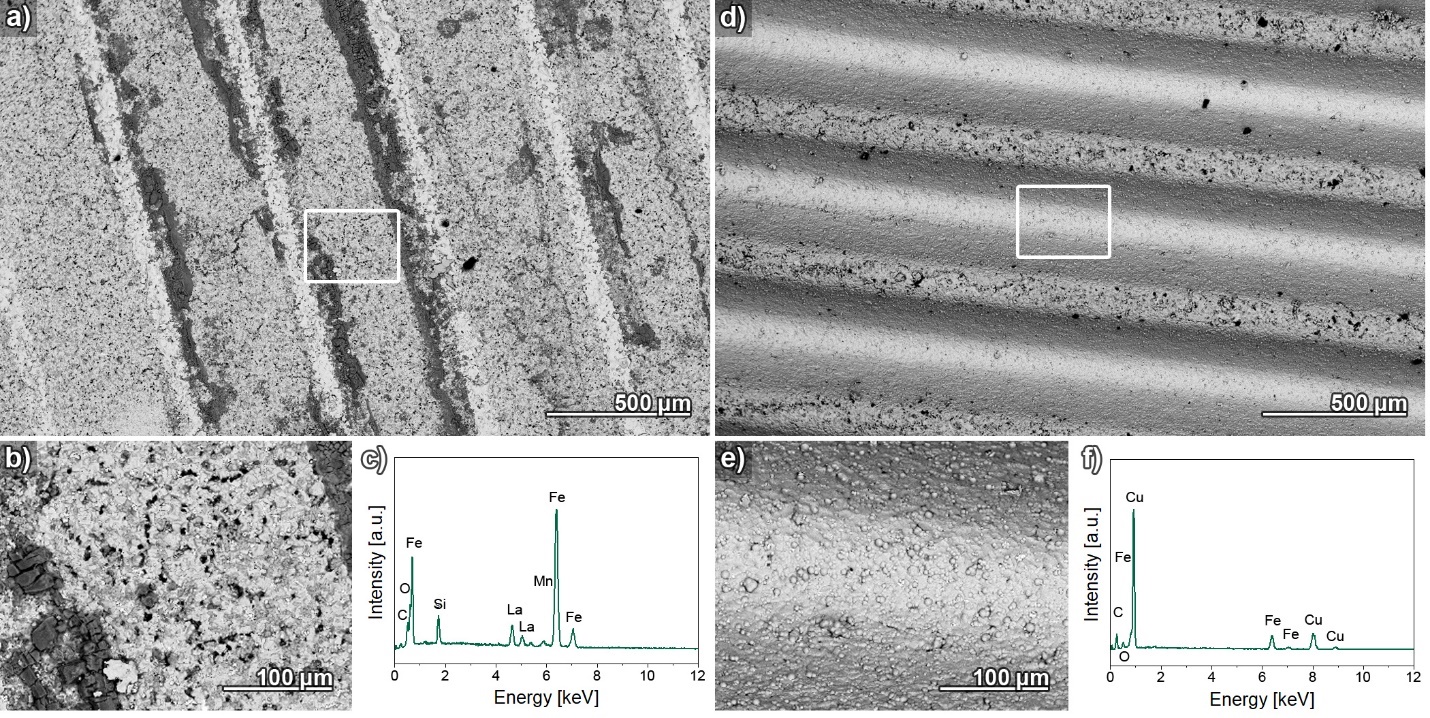


**Fig. SI 8.** Electron micrographs and representative EDX spectra of the sample aged in the commercially inspired inhibitor mixture. **a-c)** Uncoated regenerator. **d-f)** Coated regenerator.

**SEM ANALYSIS OF PLATED REGENERATOR AFTER 3 MONTHS AGING**

A microchannel subjected to an extended in-device aging duration of 3 months in the presence of an inhibitor shows an oxide-free surface (see Fig SI 9), similar to the pristine and 6-week-aged samples, corroborating the efficiency of our protection strategy. Aging experiments of even longer duration are ongoing.


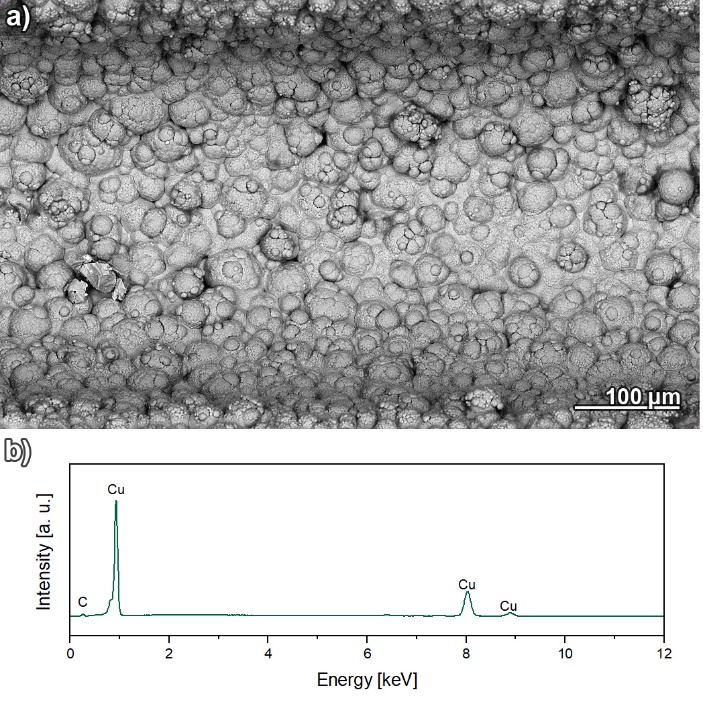


**Fig. SI 9. a)** SEM image of a copperized LaFeSi microchannel after 3 months of in-device aging in the presence of an inhibitor. **b)** Representative EDX spectrum of the channel surface shown in a), confirming the absence of oxygen.
